# Beyond Bleeding: An Analysis of Presenting Symptoms Among Black Patients with Endometrial Cancer

**DOI:** 10.1177/24731242251365480

**Published:** 2025-08-14

**Authors:** Maya E. Gross, Mindy Pike, Julianna Alson, Patrice Williams, Mollie E. Wood, Erica Marsh, Erin Carey, Til Stürmer, Ronit Katz, Whitney R. Robinson, Kemi M. Doll

**Affiliations:** ^1^ Department of Obstetrics and Gynecology, University of Washington, Seattle, Washington, USA.; ^2^ Department of Epidemiology, Gillings School of Global Public Health, University of North Carolina, Chapel Hill, North Carolina, USA.; ^3^ Department of Obstetrics and Gynecology, School of Medicine, University of Michigan, Ann Arbor, Michigan, USA.; ^4^ Department of Obstetrics and Gynecology, School of Medicine, University of North Carolina, Chapel Hill, North Carolina, USA.; ^5^ Department of Obstetrics and Gynecology, School of Medicine, Duke University, Durham, North Carolina, USA.

**Keywords:** endometrial cancer, disparities, race, fibroids, postmenopausal bleeding

## Abstract

**Objectives:**

Black patients have the highest mortality rate from endometrial cancer (EC), and yet remain underrepresented in EC research. Thus, currently published symptom patterns may not be comprehensive for this population. The purpose of this study is to analyze symptomatology among Black patients with EC in the Guidelines for Ultrasound in the Detection of Early Endometrial Cancer study and to compare with those undergoing benign hysterectomy.

**Methods:**

This is a retrospective consecutive patient sample of Black individuals undergoing hysterectomy in an academic-affiliated 10-hospital health care system from 2014 to 2020. We collected clinical, sociodemographic, and diagnostic information for 24 months before hysterectomy, using merged structured and abstracted data from electronic health records. We used descriptive statistics to describe the sample and pertinent subgroups—patients with fibroids/enlarged uteri, without postmenopausal bleeding (PMB), and patients <50 years old. Symptom distribution between subgroups was analyzed using chi-square tests and Fisher exact tests.

**Results:**

The sample included 3,455 hysterectomy patients, 12% with EC (*n* = 404). Among EC patients, 77% had PMB and 7% had no bleeding history. EC patients were symptomatic beyond bleeding, with fibroids/enlarged uteri (70%), pelvic/abdominal pain (38%), anemia (30%), and more. Young patients (8% of all EC cases) had more pelvic/abdominal pain (61% vs. 36%, *p* = 0.017) and anemia requiring transfusion (33% vs. 6%, *p* < 0.001) compared with older patients. Subgroup symptom presentations overlapped between those with and without cancer, with few symptoms differing by >20%.

**Conclusions:**

PMB alone is rarely the only presenting symptom among Black patients with EC; symptoms overlap heavily with patients presenting with benign disease. Young patients, those with fibroids/enlarged uteri, and those without PMB represent subgroups with nuanced presentations, for whom EC should be considered.

## Introduction

With rising incidence and mortality rates, endometrial cancer (EC) is the most common gynecological cancer in the United States, with the greatest annual percent increase in mortality of any cancer affecting females.^1,2^ In terms of cancer health equity, EC is emerging as one of the most critical sites of racial inequity in the United States. The Black–White mortality disparity in EC is now greater than twofold—a 100% higher mortality for Black individuals who are diagnosed.^3–8^ This is a greater disparity than that seen in breast, cervical, or colon cancers and surpasses most inequities seen in chronic diseases such as diabetes.^7–11^ In addition, the disease-specific mortality gap for Black individuals with EC in the United States is widening, in contrast to stable or decreasing mortality gaps by race in other disease sites.^1,7,8,11–13^ Advanced stage at diagnosis is one modifiable factor associated with poor prognosis among Black patients.^1,14–19^ While advanced stage at diagnosis is multifactorial, delayed symptom presentation and evaluation have been identified as a leading contributor.^19–22^

In cancer, issues of diagnostic equity include reduced uptake of cancer screenings, follow-up after abnormal screening, disclosure and evaluation of symptoms, and delays in reaching care.^10,19,23–31^ In the absence of effective screening programs for EC, diagnosis relies on detection, reporting, and timely evaluation of concerning symptoms. National guidelines for the evaluation of patients at risk for EC emphasize postmenopausal bleeding (PMB) as the key symptom to trigger diagnostic evaluation.^32,33^ Studies informing these guidelines are based largely on postmenopausal White and Asian populations, groups which experience lower rates of both incidence and mortality from EC compared with Black patients. Existing literature suggests that Black patients with EC may have different presenting symptomatology compared with non-Hispanic White patients, however, the specifics of symptom patterns, including overlap with benign gynecological symptoms, have not been clearly elucidated.^26,28,34^ In addition, as in other medical conditions, evidence suggests that in EC, even when symptoms are disclosed, Black patients are at higher risk of care delays and are less likely to be prescribed guideline-concordant care.^19,26,29,35–37^ These findings raise concerns about equitable history-taking in Black patients and others at high risk for EC, as well as in Black patients with other cancers.^19,26,38–46^ In addition, rising rates of EC among premenopausal individuals underscore the need for more enhanced understanding of EC presentations and diagnosis in this understudied subgroup.^8,47^ Beyond PMB, little is known about the symptom profile experienced by Black patients with EC before treatment.

With few exceptions, surgical treatment with hysterectomy is the standard of care for EC.^48,49^ The Guidelines for Ultrasound in the Detection of Early Endometrial Cancer (GUIDE-EC) study was established to investigate the performance of current diagnostic guidelines for EC among Black individuals undergoing hysterectomy, with an aim to develop a more effective and equitable strategy for evaluating patients presenting with potential symptoms of EC. Using GUIDE-EC data, we present an analysis of clinical signs and symptoms among Black patients undergoing hysterectomy. By comparing symptom presentations between patients with benign and malignant disease and including detailed descriptions of symptoms among younger patients and those with fibroids, we aim to expand the limited available knowledge about symptom presentations for Black patients at risk for EC.

## Materials and Methods

For the GUIDE-EC study, we employed a federated search of electronic health information and administrative data from a large academic health system, which included data from 10 hospitals and hundreds of affiliated practices, for all Black patients undergoing hysterectomy during the study period (2014–2020). Professional abstractors queried the database for structured clinical data (procedures and dates) and supplemented this with free text and imaging reports from the electronic health record (EHR). This multistep process is detailed in the methods and Supplementary Data of published prior work.^38,44,50^ Clinical, sociodemographic, and diagnostic process information was collected.

The GUIDE-EC data set is made up of all North Carolina State residents over 18 years who self-identified as Black or African American and had undergone hysterectomy in the described health system between April 4, 2014, and December 31, 2020. Individuals were excluded if they were pregnant at the time of surgery, had breast cancer diagnoses, cancers where vaginal bleeding would be nonuterine in origin (vulva, vagina, cervix), or placental-site cancers. Incarcerated individuals were also excluded, as they represent a protected class not covered by our IRB approval. Records were excluded if the hysterectomy occurred within 3 months of the EPIC go-live date (the date at which time the current electronic medical record was launched) at the surgical site to ensure adequate capture of preoperative events.

All patients from the GUIDE-EC database were included for this analysis. We aimed to describe comprehensive symptom presentations among all patients, with subgroup analyses. Identified subgroups included the presence or absence of EC, age (with cutoff of 50 years of age at the time of hysterectomy), history of or absence of fibroids or enlarged uterus, and the presence or absence of documented PMB in patients ≥50 years old.

Demographic data included date of birth, age at hysterectomy, employment status, and insurance type at the time of hysterectomy. Clinical variables included height and weight (used to calculate body mass index (BMI), date of surgery, surgical facility, presence of another malignant neoplasm of the pelvis at time of hysterectomy, and specified physician-billed and hospital-billed diagnostic and procedure Common Procedural Terminology codes within 24 months prior to hysterectomy. We captured all variables required for the Charlson comorbidity index, a validated index to classify prognostic comorbidity, at the time of hysterectomy (Table 1).^51^ Additional clinical history information, such as reported symptoms, previous diagnoses, sequelae of clinical signs and symptoms, family history, smoking history, and mental health diagnoses, was also abstracted using our EHR abstraction tool and included in the analysis (Tables 1, 2). Symptoms were documented as present, reported absent, or absent from the record.

**Table 1.** Guidelines for Ultrasound in the Detection of Early Endometrial Cancer Sample Demographics: Black Patients Undergoing Hysterectomy in a Large Health Care System from 2014 to 2020

	Endometrial cancer (*n* = 404)	No endometrial cancer (*n* = 3,051)	Overall (*n* = 3,455)
Age at hysterectomy	65.1 (58.8, 71.0)	45.6 (41.1, 50.5)	46.6 (41.7, 53.6)
Gender-expansive identity^a^	<5 (<1.2)	>15 (>0.5)	17 (0.5)
BMI (kg/m^2^)	34.9 (30.4, 42.6)	33.4 (28.6, 39.3)	33.6 (28.8, 39.6)
BMI			
≤30 kg/m^2^	70 (17.3)	773 (25.3)	843 (24.4)
31–40 kg/m^2^	147 (36.4)	1,124 (36.8)	1,271 (36.8)
>40 kg/m^2^	187 (46.3)	1,154 (37.8)	1,341 (38.8)
Smoking			
Never	268 (66.3)	2,126 (69.7)	2,394 (69.3)
Past	96 (23.8)	451 (14.8)	547 (15.8)
Current	39 (9.7)	447 (14.7)	486 (14.1)
Family history of cancer			
Breast	78 (19.3)	755 (24.8)	833 (24.1)
Ovarian	20 (5.0)	179 (5.9)	199 (5.8)
Uterine	15 (3.7)	103 (3.4)	118 (3.4)
Cervical	10 (2.5)	56 (1.8)	66 (1.9)
Mental health diagnosis			
Depression	40 (9.9)	433 (14.2)	473 (13.7)
Bipolar	<5 (<1.2)	>46 (>1.5)	53 (1.5)
Anxiety	56 (13.9)	457 (15.0)	513 (14.8)
PTSD	<5 (<1.2)	>25 (>0.8)	28 (0.8)
Comorbidity index (mean, SD)	1.4 (2.3)	0.6 (1.5)	0.66 (1.63)
0	196 (48.5)	2,286 (74.9)	2,482 (71.9)
1	105 (26.0)	462 (15.2)	567 (16.4)
2+	103 (25.5)	302 (9.9)	405 (11.7)
Insurance			
Agency	<5 (<1.2)	>3 (>0.1)	6 (0.2)
Private	137 (33.9)	2,164 (70.9)	2,301 (66.6)
Medicaid	26 (6.4)	327 (10.7)	353 (10.2)
Medicare	218 (54.0)	279 (9.1)	497 (14.4)
Tricare	<5 (<1.2)	>70 (>2.3)	74 (2.1)
Missing	19 (4.7)	205 (6.7)	224 (6.5)
Facility			
Academic hospital	327 (80.9)	590 (19.3)	917 (26.5)
Academic community hospital affiliate	77 (19.1)	2,461 (80.7)	2,538 (73.5)

Values listed as median (IQR) or *N* (%).

^a^ Indicates a trans man, gender nonbinary, or gender-queer individual as capture by indication for hysterectomy.

**Table 2.** Clinical Signs and Symptoms Associated with Endometrial Cancer Diagnosis

Symptom	EC (404, 11.7%)	No EC (3,051, 88.3%)	*p* value	FDR *p* value
Any bleeding (PMB or AUB)	376 (93.1)	2,327 (76.3)	<0.001	<0.001
PMB^a^	310 (76.7)	206 (6.8)	<0.001	<0.001
Other bleeding only	66 (16.3)	2,121 (69.5)	<0.001	<0.001
Pelvic/abdominal pain	155 (38.4)	1,576 (51.7)	<0.001	<0.001
Fatigue/light-headed	93 (23.0)	712 (23.3)	0.026	0.027
Urinary symptoms	71 (17.6)	618 (20.3)	<0.001	<0.001
Bulk symptoms	80 (19.8)	789 (25.9)	<0.001	<0.001
Menopausal symptoms	199 (49.3)	312 (10.2)	<0.001	<0.001
Sign				
BMI <30	70 (22.2)	773 (31.4)	0.001	0.001
Fibroids	239 (59.2)	2,405 (78.8)	<0.001	<0.001
Enlarged uterus	153 (37.9)	1,465 (48.0)	<0.001	<0.001
Anemia	121 (30.0)	1,406 (46.1)	<0.001	<0.001
Abnormal Pap	107 (26.5)	641 (21.0)	<0.001	<0.001
Endometrial hyperplasia	69 (17.1)	81 (2.7)	<0.001	<0.001
Transfusion history	35 (8.7)	276 (9.1)	0.801	0.801

Values listed as *N* (%); chi-square tests were used to test differences between EC/no EC groups; FDR *p* value is calculated using the Benjamini–Hochberg method.

^a^ Postmenopausal bleeding (PMB) includes patients with postmenopausal bleeding alone, as well as patients who had postmenopausal bleeding documented in addition to other abnormal bleeding.

EC, endometrial cancer; AUB, abnormal uterine bleeding; FDR, false discovery rate.

We collected granular information on select clinical signs and symptoms common among patients undergoing hysterectomy, if present at the time of or within a 30-day window of the initial diagnostic procedure. We characterized bleeding pattern as PMB or “other bleeding.” Patients were categorized as having PMB if bleeding had been documented in the chart exclusively as postmenopausal, or if bleeding had been documented as postmenopausal in addition to other bleeding. “Other bleeding” was defined as any abnormal uterine bleeding (AUB) not specifically documented as postmenopausal, regardless of age. Examples of these designations included AUB, dysfunctional uterine bleeding, menometrorrhagia, menorrhagia, polymenorrhea, anovulation, breakthrough bleeding, bleeding during intercourse, bleeding with pelvic pressure, and postcoital spotting. Pain reported by patients in any pelvic or abdominal structure was grouped into pelvic/abdominal pain. Urinary symptoms included frequency, retention, or incontinence. Menopausal symptoms were included if attributed to menopause in the documentation; select examples include vasomotor symptoms, mood or energy changes, among others. Bulk symptoms included the specific notation of “bulk symptoms” in clinical notes, as well as constipation, bloating, and pelvic pressure. A cut-point for BMI at 30 kg/m^2^ was included as a clinical sign for analysis.^52^ History of fibroids, enlarged uterus, anemia, history of abnormal Pap, history of endometrial hyperplasia, and transfusion history were included as clinical signs if documented as existing or historical problems in clinical progress notes.

### Statistical Analyses

We used descriptive statistics including median (IQR) and *n* (%) to evaluate demographic factors, signs, and symptoms overall and for important subgroups. First, we stratified by EC status, overall and among those with fibroids and/or enlarged uterus. Then, among EC cases, we evaluated three important less common presentations. We stratified by age to highlight symptom presentation in those with EC under 50 years old, and we stratified by PMB to characterize those without the most common presenting symptom of EC. We additionally described symptoms among patients with EC stratified by a history of fibroids or enlarged uterus. A final sensitivity analysis examined clinical signs/symptoms by age using a cutoff of 51.4 years, as this is the median age of natural menopause among Black women in the United States (Supplementary Table S1).^53^ Chi-square and Fisher’s exact tests were used to compare proportions. To account for multiple comparisons, false discovery rate (FDR)-adjusted *p*-values were calculated using the Benjamini–Hochberg method and an FDR *p*-value of <0.05 was considered.^54^ Statistical analyses were conducted using Stata Version 18.0 (StataCorp LLC, College Station, TX).^55^ Differences >20% between groups were highlighted as clinically meaningful/significant differences.

## Results

The initial dataset included 3,887 individuals. Exclusion criteria were applied as described. During the data cleaning process, records were excluded for reasons such as empty data files (records with no data entry), records erroneously coded with hysterectomy, and hysterectomies conducted at facilities outside the health care system network, and files missing after the data transfer were excluded (Supplementary Fig. S1). The final dataset comprised 3,455 Black individuals.

Patient characteristics are summarized in Table 1, stratified by diagnosis of EC. A total of 404 patients (12%) had an EC diagnosis; of patients with cancer, 8% (*n* = 33) were <50 years of age at the time of hysterectomy. The age range of patients with EC was 27.4–89.8 years. We did not restrict by gender identification and there were a small number (*N* = 17) of nonbinary and trans people who met all the inclusion criteria. Mental health diagnoses were notable for depression (14%) and anxiety (15%), consistent with national estimates.^56^ Most participants (67%) were enrolled in commercial health plans, with 10% enrolled in Medicaid and 14% in Medicare plans. On final pathology, 39% of patients in both EC (*n* = 156) and benign (*n* = 1177) had a diagnosis of adenomyosis. In no cases of EC was adenomyosis the documented reason for hysterectomy.

Table 2 reports preoperative clinical signs and symptoms by EC status. Patients were highly symptomatic in both groups, and there was significant overlap in symptom presentation between patients with and without EC, but there were statistically significant differences in the rates of most symptoms due to the large sample size and ability to detect small differences. The vast majority of patients with EC experienced bleeding symptoms: 77% had documented PMB, 16% of patients with EC had other bleeding only, and 7% had no documented bleeding symptoms. Patients without EC had less bleeding symptoms overall, and were more likely to have bleeding documented as other than postmenopausal (69% vs. 16%, *p* < 0.001). Patients with EC had higher rates of menopausal symptoms (49% vs. 10%, *p* < 0.001), slightly lower rates of pelvic/abdominal pain (38% vs. 52%, *p* < 0.001), and reported similar rates of fatigue/lightheadedness (23.0% vs. 23.3%, *p* = 0.027). Fibroids were present in more than half the patients in both groups but were more common in patients without EC (59% vs. 79%, *p* < 0.001). Patients in both groups suffered from anemia (30% vs. 46%, *p* < 0.001).

Among patients with EC, age was associated with distribution of clinical signs and symptoms (Supplementary Table S1). Nearly all younger patients with EC had AUB (94%). Young patients with EC were more likely than older EC patients to have pelvic or abdominal pain (61% vs. 36%, *p* = 0.017) and anemia requiring transfusion (33% vs. 6%, *p* < 0.001). As expected, bleeding type differed by age; the majority of patients above the age of 50 had documented PMB (84%); however, 9% of patients above the age of 50 had abnormal bleeding not flagged as postmenopausal. Using an age cutoff of 51.4, results did not differ significantly (Supplementary Table S2).

We then compared symptoms between those with and without EC, restricting the group to patients <50 years old (Table 3). Young patients with EC had similar symptom presentations to those without EC. While not statistically significant due to small sample size, younger patients were less likely to endorse bulk symptoms (18% vs. 49%, *p* = 0.566). Rates of fibroids were higher in the no-EC group, but present in at least half the patients in both groups (51% vs. 81%, *p* < 0.001). Patients with EC in this younger group were numerically more likely to be anemic (70% vs. 50%, *p* = 0.200), have a history of transfusion (33% vs. 10%, *p* < 0.001), and to have a history of endometrial hyperplasia (27% vs. 1%, *p* < 0.001).

**Table 3.** Endometrial Cancer Versus No Endometrial Cancer in Patients Under 50

Symptom	EC <50 years (33, 1.5%)	No EC <50 years (2,215, 98.5%)	*p* value	FDR *p* value
Any bleeding (PMB or AUB)	31 (93.9)	1,808 (81.6)	0.203	0.435
PMB^a^	0 (0.0)	8 (0.4)	1.000	1.000
Other bleeding only	31 (93.9)	1,800 (81.3)	0.070	0.210
Pelvic/abdominal pain	20 (60.6)	1,160 (52.4)	0.652	0.752
Fatigue/light-headed	11 (33.3)	540 (24.4)	0.235	0.441
Urinary symptoms	8 (24.2)	421 (19.0)	0.760	0.814
Bulk symptoms	6 (18.2)	564 (49.0)	0.453	0.566
Menopausal symptoms	<5 (<15.0)	121 (5.5)	0.030	0.113
Clinical Sign				
BMI <30	<5 (<15.0)	545 (30.7)	0.250	0.417
Fibroids	17 (51.5)	1,804 (81.4)	<0.001	<0.001
Enlarged uterus	14 (42.4)	1,114 (50.3)	0.369	0.554
Anemia	23 (69.7)	1,106 (49.9)	0.080	0.200
Abnormal Pap	10 (30.3)	482 (21.8)	0.449	0.612
Endometrial hyperplasia	9 (27.3)	23 (1.0)	<0.001	<0.001
Transfusion history	11 (33.3)	221 (10.0)	<0.001	<0.001

Values listed as *N* (%); Fisher’s exact tests were used to test differences between groups; FDR *p* value is calculated using the Benjamini–Hochberg method.

^a^ Postmenopausal bleeding includes patients with postmenopausal bleeding alone, as well as patients who had postmenopausal bleeding documented in addition to other abnormal bleeding.

EC, endometrial cancer; AUB, abnormal uterine bleeding; FDR, false discovery rate.

Among those with EC over 50 years old, we assessed overall symptoms and compared symptom profile differences between those with documented PMB (84%) and those without (Table 4). More than half the patients without documented PMB (57%) had documented “other bleeding,” nearly half (47%) experienced pelvic or abdominal pain, and more than one-third (34%) had fatigue/lightheadedness. The majority had a history of fibroids (64%), and nearly one-third (31%) had a history of anemia. Compared with patients with PMB, those without documented PMB had lower reports of menopausal symptoms (<9% vs. >62%, *p* < 0.001), and higher rates of fatigue-lightheadedness (34% vs. 20%, *p* = 0.028), but otherwise had very similar symptom presentations.

**Table 4.** Symptom Presentation Among Endometrial Cancer Patients over 50, Overall and by PMB

Symptom	Overall (*n* = 371)	PMB (*n* = 310)	No PMB (*n* = 61)	*p* Value	FDR *p* value
Any bleeding (PMB or AUB)	345 (93.0)	310 (100.0)	35 (57.4)	<0.001	<0.001
PMB^a^	310 (83.6)	310 (100.0)	0 (0.0)	NA	NA
Other bleeding only	35 (9.4)	0 (0.0)	35 (57.4)	NA	NA
Pelvic/abdominal pain	135 (36.4)	106 (34.2)	29 (47.5)	0.001	0.004
Fatigue/light-headed	82 (22.1)	61 (19.7)	21 (34.4)	0.013	0.028
Urinary symptoms	63 (17.0)	54 (17.4)	9 (14.8)	0.478	0.690
Bulk symptoms	74 (19.9)	54 (17.4)	20 (32.8)	0.003	0.009
Menopausal symptoms	198 (53.4)	>193 (>62.3)	<5 (<8.2)	<0.001	<0.001
Sign					
BMI <30	66 (22.5)	47 (19.3)	19 (38.8)	0.005	0.013
Fibroids	222 (59.8)	183 (59.0)	39 (63.9)	0.387	0.629
Enlarged uterus	139 (37.5)	115 (37.1)	24 (39.3)	0.258	0.479
Anemia	98 (26.4)	79 (25.5)	19 (31.2)	0.626	0.678
Abnormal Pap	97 (26.2)	83 (26.8)	14 (23.0)	0.731	0.731
Endometrial hyperplasia	60 (16.2)	52 (16.8)	8 (13.1)	0.515	0.669
Transfusion history	24 (6.5)	19 (6.1)	5 (8.2)	0.548	0.648

Values listed as *N* (%); Fisher’s exact and chi-square tests were used to test differences between groups; FDR *p* value is calculated using the Benjamini–Hochberg method.

^a^ Postmenopausal bleeding includes patients with postmenopausal bleeding alone, as well as patients who had postmenopausal bleeding documented in addition to other abnormal bleeding.

AUB, abnormal uterine bleeding; FDR, false discovery rate.

Finally, we evaluated symptom presentation patterns among those with fibroids or an enlarged uterus (80% *N* = 2,768) and EC, to determine whether there were specific distinguishing features of EC in this group, which differed from the overall sample (Supplementary Table S3). Overall, symptom distributions were largely similar with and without EC diagnosis. The few notable differences were heavily dependent on age-related factors: PMB (76% vs. 6%, *p* < 0.001) and menopausal symptoms (49% vs. 10%, *p* < 0.001). Otherwise, anemia diagnosis was more common in the non-EC group (50% vs. 30%, *p* < 0.001), although transfusion history was similar (10% vs. 9%, *p* = 0.568). As expected, endometrial hyperplasia history was more common in those with EC. Symptoms among patients with fibroids and EC were similar to those in the entire sample.

## Discussion

Black patients in the United States have been chronically underrepresented in consensually conducted research efforts, which has led to an imbalanced understanding of the root causes of disease and treatment responses across a wide range of health conditions.^3,57–60^ EC is no exception to this inequity. Black patients with EC experience delays in diagnosis, which contribute to the advanced stage at diagnosis and inferior cancer outcomes, yet little is known about the root cause of these disparities. In this analysis of the GUIDE-EC dataset, we present the largest published examination of clinical signs and symptoms among Black individuals with EC, utilizing comprehensive inpatient and outpatient data to compare symptoms in patients with benign disease. In addition, we present analyses of symptoms among patients without documented PMB, those under the age of 50, and those with a history of fibroids or enlarged uteri—groups in which diagnosis may be less straightforward than in postmenopausal patients with classical PMB. Our findings highlight the diverse patient and symptom composition within the larger group of Black patients with EC and emphasize overlapping symptom presentations between benign and malignant disease.

Most cases of EC are diagnosed in postmenopausal patients, but the definition and recognition of the postmenopausal state can be missed at both the patient and provider levels. While the median age of the final menstrual period in the United States is 51, the menopausal transition can last for years, with fluctuating symptoms and bleeding patterns.^61,62^ In a qualitative study evaluating the prediagnostic experiences of Black women with EC, misattribution of bleeding on the part of both patients and providers was widespread.^19^ The complex symptom presentation in our sample highlights that despite experiencing higher rates and severity of symptoms related to EC diagnoses, Black individuals are more likely to have symptom codes of “abnormal” as opposed to PMB, and that providers often underemphasize or brush off reported episodes of abnormal and PMB in Black women.^14,19,26,39^ Black patients are additionally less likely than White patients to receive guideline-concordant care after reporting PMB.^18,19^ In our study, 84% of people with EC over the age of 50 had bleeding explicitly documented as PMB, and most had additional signs and symptoms. This mischaracterization, whether introduced by patients or providers, has important implications regarding bleeding evaluation and public health messaging about EC. By asking solely about PMB, we may miss or delay a diagnosis in more than 15% of patients with EC.

Rates of EC among patients <50 years of age are rising, and providers are likely to encounter more patients with risk factors for EC at a young age.^8,47^ Specific risk factors for young patients with EC are well documented, but existing literature on symptomatology in this group is sparse, centering on abnormal bleeding.^32^ In this study, we highlighted that young patients with EC are more likely than older EC patients to report pelvic/abdominal pain, have significant anemia, and a transfusion history. Young patients with EC experienced similar symptom profiles compared with their counterparts with benign disease. This symptom overlap raises concerns about relying on symptom tropes to distinguish between benign and malignant disease; consideration of earlier evaluation with tissue sampling in this group is warranted if presenting with symptoms.

In our study, fibroids or enlarged uteri coexisted in 70% of patients with EC. Fibroids and enlarged uteri can cause irregular bleeding throughout a patient’s reproductive lifetime. The presence of fibroids is known to reduce the efficacy of transvaginal ultrasound (TVUS) in the diagnosis of EC and can challenge the interpretation of symptoms by both patients and providers, yet guideline-informing studies have largely excluded patients with fibroids.^19,40,43,44,63^ There is a paucity of data in the literature examining the impact of fibroids diagnostic pathways for EC. For patients with fibroids, we demonstrated that distinguishing between benign and malignant disease, based on presenting symptoms alone, is unreliable. With this knowledge in hand, providers must not abandon an evaluation of EC based on personal and clinical characteristics, such as perimenopause or history of fibroids; in doing so, we have the potential to harm our patients, prolonging their time to definitive diagnosis and undermining their trust.

Our dataset highlights that PMB alone is rarely the only presenting symptom among Black EC patients undergoing hysterectomy. This study adds to the growing body of research spotlighting the inadequacies of current diagnostic processes. For clinicians, it is imperative to include EC in the differential diagnosis of Black patients with abnormal bleeding regardless of coexistent longer term symptoms, to avoid attributing symptoms to benign disease without excluding the possibility of neoplasia or malignancy, and to have a low threshold for tissue sampling or other evaluations for EC in this group. For researchers, based on the findings from the GUIDE-EC study, it is imperative to continue prioritizing the development of new evaluation and diagnostic tools, which narrow rather than widen disparities for Black patients and other patients with atypical EC presentations.

### Strengths and Limitations

The strengths of this study include the large sample size with nearly 3,500 individuals. Detailed data abstraction, performed by abstraction experts, allowed us to chronicle the diagnostic journey for Black patients experiencing AUB. Data from multiple hospitals and clinics, in addition to scanned reports from outside the health care system, allowed us to overcome the limitations of single-institution data to reflect care that often crosses between different health care entities. In addition, we present detailed portraits of subgroups underrepresented in EC symptomatology research.

This work was conducted exclusively among Black patients with EC, who have been largely excluded from previous research on EC. In previous studies that included largely non-Black populations, the prevalence of PMB among patients with EC was 90%, with a 9% rate of EC among patients with PMB.^64^ Addressing this gap in research is important, and our design to focus on Black individuals was to highlight potential differences and nuances within this group that can be lost when comparisons between racial groups are emphasized. Despite this strength, the omission of a comparison group does limit the direct within-study comparison in this large health care system.

Inability to formally assess menopausal status was a necessary limitation of our data, as this metric was not reliably documented in the medical records; interpretation of bleeding as postmenopausal or not was dependent on provider documentation. We used two separate age cutoffs for our analyses, specifically at age 50 and at age 51.4, which has been described as the natural median age of menopause among Black patients in the United States, as a proxy for menopausal status.^53^ We advocate for a more formal and standardized emphasis on clear documentation of menopausal status as a potential solution to this problem; existing literature demonstrates that both patients and providers can misattribute PMB to other causes if not formally documented, which can create harm via false reassurance.^19,20,65^ In a previous study of Black and White women with Medicare insurance, we found that Black women, all over the age of 67, were more likely to have their bleeding categorized as a “heavy period” rather than postmenopausal, which led to the absence of guideline-adherent evaluation.^18^ Formal menopausal documentation can mitigate this pattern.

For fibroid size, for this analysis, we used enlarged uterine size as a proxy for clinically significant fibroids. This may also be a rough proxy for adenomyosis, which was present at an equal rate in both benign and EC cases. As this article focuses on initial symptom presentation, we focused on signs and symptoms clear from an initial clinician visit. Information on fibroid size (and location) and often the presence of adenomyosis are all determined *after* the choice of diagnostic work (i.e., with a TVUS). We do acknowledge that failure to abstract data on adenomyosis independently was a limitation of our abstraction protocol, although our focus was on initial presentation and the discernment between EC and all potential benign causes. Additionally, we do not have or include data on histological subtype or molecular classification of tumors. We agree that further research should investigate these variables for interest in how these factors may influence presentation.

Our sample identifies individuals based on hysterectomy, which selects for different symptom severity in those with benign and malignant disease. For benign disease, included patients are more likely to have severe symptoms not amenable to conservative management. For EC, our included patients are less likely to have grossly advanced-stage disease and/or high burden of comorbidities that may preclude hysterectomy. Consequently, differences in symptomatology between those with benign disease (relatively more severe) and those with EC undergoing hysterectomy (relatively less severe) may be less pronounced than in an “all-comer” presurgical clinical population. This would bias our results in underestimating the differences between those with and without EC. However, our findings demonstrate a heterogeneous patient population in both benign and malignant disease, with more symptom overlap than differences, which makes meaningful clinical distinction based on symptoms challenging.

## Health Equity Implications

A universally applied approach to diagnosis based on a low-risk population does not equitably serve patients with a different set of risk factors for disease severity. This was borne out of menopause research, where exclusion of Black patients from the Study of Women’s Health Across the Nation led researchers to miss a signal that Black patients may experience earlier menopausal symptoms.^53^ Without understanding symptom presentation in our most high-risk groups, we similarly cannot begin to address disparities in the diagnosis of EC. When evaluating patients with symptoms of gynecological disease, providers use clinical guidelines, in conjunction with available evidence on differential diagnoses, to guide practice. For EC, the body of evidence has been dominated by an investigation into the presence of PMB; this narrow focus for triggering diagnostic evaluation for EC may mislead clinicians into expecting relatively mild gynecological symptoms otherwise. Our study highlights that the presentation of EC in Black patients is variable, complex, and less well defined than previously thought, and that PMB alone is rarely the only presenting symptom among Black EC patients undergoing hysterectomy. Clinicians of all specialties must include EC in the differential diagnosis of Black patients with abnormal bleeding, regardless of coexistent longer term symptoms.

## Abbreviations Used

AUB: Abnormal uterine bleeding
BMI: Body mass index
EC: Endometrial cancer
EHR: Electronic health record
FDR: False discovery rate
GUIDE-EC: Guidelines for ultrasound in the detection of early endometrial cancer
PMB: Postmenopausal bleeding
TVUS: Transvaginal ultrasound
