# Supplementary material for: Beyond Bleeding: An Analysis of Presenting Symptoms Among Black Patients with Endometrial Cancer
Source: Health Equity. 2025 Aug 14;9(1):375–85. doi: 10.1177/24731242251365480 (PMC12412387; doi:10.1177/24731242251365480)
Supplement: Supplementary Data [file 24731242251365480_supplementary_data.docx]

**Supplemental Files**

**Supplemental Figure 1: CONSORT diagram for GUIDE-EC Symptom Analysis**


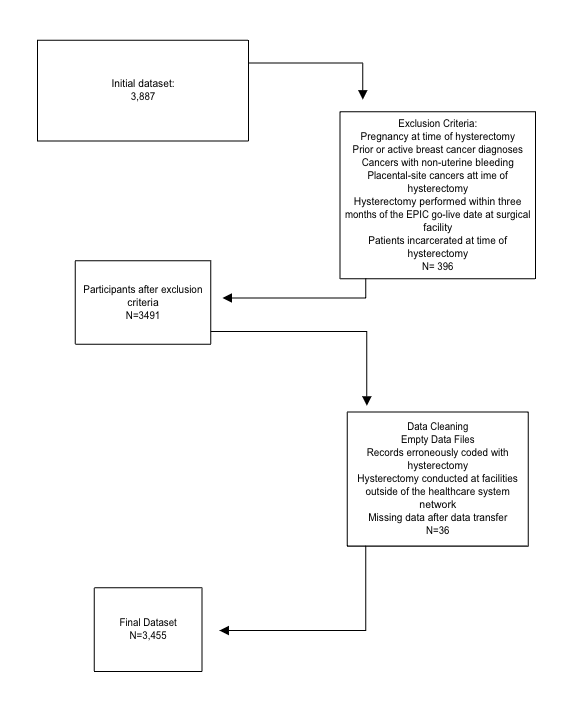


**Supplemental Table 1: Clinical signs and symptoms by age in individuals with Endometrial Cancer**

| **Symptom** | Age <50y (33, 8.2%) | Age ≥50y (371, 91.8%) | p-value | FDR p-value |
| --- | --- | --- | --- | --- |
| Any bleeding (PMB or AUB) | 31 (93.9) | 345 (93.0) | 0.533 | 0.615 |
| PMB* | 0 (0.0) | 310 (83.6) | <0.001 | <0.001 |
| Other bleeding only | 31 (93.9) | 35 (9.4) | <0.001 | <0.001 |
| Pelvic/abdominal pain | 20 (60.6) | 135 (36.4) | 0.007 | 0.017 |
| Fatigue/Light-headed | 11 (33.3) | 82 (22.1) | 0.048 | 0.103 |
| Urinary symptoms | 8 (24.2) | 63 (17.0) | 0.102 | 0.191 |
| Bulk symptoms | 6 (18.2) | 74 (19.9) | 0.406 | 0.507 |
| Menopausal symptoms | <5 (<15.0) | >195 (>52.6) | <0.001 | <0.001 |
| **Clinical Sign** |  |  |  |  |
| BMI <30 | <5 (<15.0) | >65 (>17.5) | 0.794 | 0.850 |
| Fibroids | 17 (51.5) | 222 (59.8) | 0.118 | 0.196 |
| Enlarged uterus | 14 (42.4) | 139 (37.5) | 0.874 | 0.874 |
| Anemia | 23 (69.7) | 98 (26.4) | <0.001 | <0.001 |
| Abnormal pap | 10 (30.3) | 97 (26.2) | 0.233 | 0.349 |
| Endometrial hyperplasia | 9 (27.3) | 60 (16.2) | 0.257 | 0.350 |
| Transfusion history | 11 (33.3) | 24 (6.5) | <0.001 | <0.001 |
| Note: Values listed as N (%); Fisher’s exact tests were used to test differences between groups; FDR p-value is calculated using the Benjamini-Hochberg method  *Postmenopausal bleeding includes patients with postmenopausal bleeding alone, as well as patients that had postmenopausal bleeding documented in addition to other abnormal bleeding | | | | |

**Supplemental Table 2. Clinical Signs and symptoms by age in EC (using cutoff of 51.4)**

| **Symptom** | Age <51.4y (36, 8.9%) | Age ≥51.4y (368, 91.1%) | p-value | FDR p-value |
| --- | --- | --- | --- | --- |
| Any bleeding (PMB or AUB) | 34 (94.4) | 342 (92.9) | 0.647 | 0.693 |
| PMB* | <5 (<13.9) | 309 (84.0) | <0.001 | <0.001 |
| Other bleeding only | >30 (>88.2) | 33 (9.0) | <0.001 | <0.001 |
| Pelvic/abdominal pain | 21 (58.3) | 134 (36.4) | 0.021 | 0.052 |
| Fatigue/Light-headed | 11 (30.6) | 82 (22.3) | 0.073 | 0.136 |
| Urinary symptoms | 9 (25.0) | 62 (16.9) | 0.143 | 0.238 |
| Bulk symptoms | 8 (22.2) | 72 (19.6) | 0.194 | 0.291 |
| Menopausal symptoms | <5 (<13.9) | >195 (>53.0) | <0.001 | <0.001 |
| **Sign** |  |  |  |  |
| BMI <30 | <5 (<13.9) | >65 (>17.7) | 0.616 | 0.710 |
| Fibroids | 19 (52.8) | 220 (59.8) | 0.032 | 0.068 |
| Enlarged uterus | 14 (38.9) | 139 (37.8) | 1.000 | 1.000 |
| Anemia | 23 (63.9) | 98 (26.6) | <0.001 | <0.001 |
| Abnormal pap | 11 (30.6) | 96 (26.1) | 0.428 | 0.584 |
| Endometrial hyperplasia | 9 (25.0) | 60 (16.3) | 0.481 | 0.601 |
| Transfusion history | 11 (30.6) | 24 (6.5) | <0.001 | <0.001 |
| Note: Values listed as N (%). Age 51.4 was selected as the cutoff, as the median age of natural menopause among Black patients in the U.S.; Fisher’s exact and chi-square tests were used to test differences between groups; FDR p-value is calculated using the Benjamini-Hochberg method | | | | |

Note: Values listed as N (%)

*Postmenopausal bleeding includes patients with postmenopausal bleeding alone, as well as patients that had postmenopausal bleeding documented in addition to other abnormal bleeding

**Supplemental table 3: symptom presentation among patients with fibroids or enlarged uterus (n=2768), EC vs. no EC**

| **Symptom** | EC (284, 10.3%) | No EC (2484, 89.7%) | p-value | FDR p-value |
| --- | --- | --- | --- | --- |
| Any bleeding (PMB or AUB) | 264 (93.0) | 2,034 (81.9) | <0.001 | <0.001 |
| PMB only | 217 (76.4) | 155 (6.2) | <0.001 | <0.001 |
| Other bleeding only | 47 (16.6) | 1879 (75.6) | <0.001 | <0.001 |
| Pelvic/abdominal pain | 118 (41.6) | 1302 (52.4) | <0.001 | <0.001 |
| Fatigue/Light-headed | 79 (27.8) | 596 (24.0) | 0.028 | 0.033 |
| Urinary symptoms | 54 (19.0) | 490 (19.7) | <0.001 | <0.001 |
| Bulk symptoms | 66 (23.2) | 671 (27.0) | <0.001 | <0.001 |
| Menopausal symptoms | 141 (49.7) | 251 (10.1) | <0.001 | <0.001 |
| **Sign** |  |  |  |  |
| BMI <30 | 56 (24.9) | 607 (30.2) | 0.099 | 0.107 |
| Anemia | 85 (29.9) | 1246 (50.2) | <0.001 | <0.001 |
| Abnormal pap | 72 (25.4) | 495 (19.9) | 0.002 | 0.003 |
| Endometrial hyperplasia | 49 (17.3) | 55 (2.2) | <0.001 | <0.001 |
| Transfusion history | 25 (8.8) | 245 (9.9) | 0.568 | 0.568 |
| Note: Values listed as N (%); Fisher’s exact and chi-square tests were used to test differences between groups; FDR p-value is calculated using the Benjamini-Hochberg method  *Postmenopausal bleeding includes patients with postmenopausal bleeding alone, as well as patients that had postmenopausal bleeding documented in addition to other abnormal bleeding | | | | |
